# 25 YEARS AFTER THE PASSING OF THE NPO LAW: EXAMINING THE ORGANIZATIONAL CAPACITY OF OLDER VOLUNTEER ORGANIZATIONS

**DOI:** 10.1093/geroni/igad104.3136

**Published:** 2023-12-21

**Authors:** Li-Mei Chen

**Affiliations:** George Mason University College of Public Health, Fairfax, Virginia, United States

## Abstract

Japan has experienced a growth in non-profit organizations since the enactment of the NPO Law in 1998. In 2022, there were 175,046 volunteer organizations with approximately 6.7 million volunteers. Many of these non-profits providing community-based social services are disproportionately represented by older volunteers. Tbe benefits of older volunteerism have been well-documented stating that society gains from the older adults’ contributions as well as older adults’ lives are improved and enhanced through volunteerism. However, evidence is lacking as to how older volunteer organizations have evolved in the past 25 years. This study investigates the types and characteristics of non-profit organizations which recruit older volunteers based on their organizational capacity. Surveys were mailed to 500 volunteer organizations which provide social services in Japan. 155 (Findings from cluster analysis showed that there are types of organizations ranging from “Achiever” which excelled in all dimensions of organizational capacity to “Challenged” which lacked in all dimensions. Other volunteer types include “Government dependent” type which are non-profit organizations which depend on government programs, “Autonomous” type which excel in offering role flexibility to volunteers but lack capacity in all other dimensions, and “Bonding” type which is formed by a tight-knit group of persons and are no longer seeking new volunteers. Bivariate analysis was conducted to examine the relationship between the organization types and volunteer outcomes . “Achiever” type found an increase in volunteers and rarely experienced lack of volunteers while the “Challenged” type seldomly experienced an increase and frequently experienced lack of volunteers.

